# Stability Analysis of Two Power Converters Control Algorithms Connected to Micro-Grids with Wide Frequency Variation

**DOI:** 10.3390/s22187078

**Published:** 2022-09-19

**Authors:** Jaime Rohten, Felipe Villarroel, Esteban Pulido, Javier Muñoz, José Silva, Marcelo Perez

**Affiliations:** 1 Department of Electrical and Electronic Engineering, Universidad del Bío-Bío, Av. Collao 1202, Concepción 4051381, Chile; 2 Department of Electrical Engineering, Universidad de Concepción, E. Larenas 219, Concepción 4089100, Chile; 3 Department of Electrical Engineering, Universidad Técnica Federico Santa María, Avenida España 1680, Valparaíso 2340000, Chile; 4 Department of Electrical Engineering, Universidad de Talca, Camino Los Niches Km. 1, Curicó 3340000, Chile; 5 Department of Engineering Science, Universidad de Los Lagos, km. 6, Camino A Chinquihue, Puerto Montt 5480000, Chile; 6 Department of Electronic Engineering, Universidad Técnica Federico Santa María, Avenida España 1680, Valparaíso 2340000, Chile

**Keywords:** linear control, nonlinear control, stability analysis

## Abstract

Distributed power generation, micro-grids, and networks working in islanding mode have strong deviations in voltage quantities. These deviations can be divided into amplitude and frequency. Amplitude deviations are well-known and studied, as they are common in small and big grids. However, deviations on the ac mains frequency have not been widely studied. The literature shows control schemes capable of bearing these variations, but no systematic analysis has been performed to ensure stability. As the majority of power converters are designed for big grids, their analysis and design neglect frequency disturbances, therefore those devices allow a very small frequency operating window. For instance, in power converters that need to be synchronized to the grid, the standard deviation does not go beyond 0.5 Hz, and for grid-tied inverters it does not go beyond 1 Hz, whereas variations of around 8 Hz can be expected in micro-grids. This work presents a comprehensive analysis of the control system’s stability, where two different control schemes for a back-to-back static converter topology are implemented and studied under a wide variable grid frequency. Because the behavior of power converters is nonlinear and coupled, dynamic and static decouplers are usually introduced in the controller, being a key element on the scheme according to the findings. The results show that using just a static decoupler does not guarantee stability under frequency variations; meanwhile, when a dynamic decoupler is used, the operating window can be greatly extended. The procedure shown in this paper can also be extended to other control algorithms, making it possible to carefully choose the control system for a variable frequency condition. Simulated and experimental results confirm the theoretical approach.

## 1. Introduction

Certainly, due to the proliferation of distributed generation, micro-grids, islanded systems, and non-conventional power generation sources, grid frequency variation has become a topic for research and study. These sources, for instance, in aircraft and others applications, present important changes in their operating conditions, particularly concerning frequency, which may deviate as far as 75%, as shown in [1], or even 100% as shown in [2,3,4,5]. In addition, significant fluctuations in generating conditions are normal in wind power turbines and weak power systems (−12%, +8%) [6,7,8,9], and are also present as critical frequency variation ranges for normal power systems (−6%, +4%) [10] in contrast to conventional stiff and high power grids in normal operation frequency variation ranges (±1%) [10]. In addition, power systems working in islanding mode, as well as ship power systems, are also of interest, where large variations in the fundamental frequency are part of the normal operating conditions. In addition, many aircraft power systems are designed for a wide-frequency environment in order to obtain a suitable response for these kinds of disturbances [2,3,4,5].

The increasing performance requirements placed on grid synchronization for proper converter operation under varying conditions has given rise to improved discrete Phase-Locked Loops (PLLs) schemes. Many of these synchronization methods have been developed to operate under variable frequency conditions [5,11,12,13,14,15,16,17,18,19], and can operate correctly even under both polluted [5,15,19,20,21] and unbalanced *ac* voltages [22]. They can be readily utilized in several power electronics systems’ applications, being of particular interest for grid-connected power converters such as [23,24,25,26,27,28], given that their use on these power converters is mandatory for adequate operation [1,13,19,29,30]. From the control point of view, frequency and phase synchronization is critical for synchronous reference frame (SRF)-based controllers, due to their reliance on accurate variable transformations used in the control loops [5]. Although these algorithms allow the estimation of the grid frequency with a good degree of accuracy [1,2,3,4,5,6,7,8,9,10,11,12,13,14], the effects of a variable grid frequency are often neglected or not explicitly considered in the design of the power converter control loops [31,32]. However, when weak power systems are considered, or when the system is operating in islanding mode, variation in the system frequency is to be expected [1,2,3,4,5,8,9].

Regarding the design of power converter control loops, the most common scheme, used for instance on active rectifiers, is designed as a master loop controlling the *dc* link voltage and power factor, which gives the current or power reference to an inner control loop. However, due to the high degree of coupling in the inner loop, decouplers are mandatorily included in order to induce a single-input and single-output behavior. Decouplers can be classified as static [33] and dynamic [34,35,36]. *Static decouplers* are a simple and appealing alternative because sensors and mathematical calculations are reduced, leading to a lowered computational effort. This is an important feature, especially in multilevel converters where the controllers have a higher complexity. On the other hand, *dynamic decouplers* have also been widely used in power electronics applications because they have a better performance and behavior, but with additional computational costs and the need to sense more variables and estimate the system’s parameters.

The typical approach used to control grid-connected power converters is to consider a constant grid frequency [32,34,35,36,37], meaning that a *static decoupler* is implicitly adopted; however, if the grid frequency is not constant, the model ceases to represent the true system dynamics. For instance, controller design of three-phase power converters is usually carried out in a rotating *dq* reference frame. During this process, the electrical system’s frequency is typically considered as a fixed parameter. Then, controller tuning is performed to achieve the desired performance specifications, such as settling time, damping factor, and, most importantly, stability. However, after the controller design is complete, its parameters are left fixed, thus neglecting the significant variation in the electrical system’s frequency that can be present during operation. Therefore, a systematic study of the effect of frequency variation on the behavior of the control system is required. In this way, the issue of variable grid frequency can determine when the controllers, based on *dynamic* or *static decoupler*, are valid, or, more strictly, stable in a wide variable frequency environment.

This paper presents a comprehensive analysis of the stability of the control system in one of the most common grid-tied converters under variations in grid frequency. As cases of study, this work studies the behavior of the most commonly used *dq* SRF-based control methods as applied in three-phase grid-connected boost rectifiers, obtaining how far the frequency may deviate from design values without affecting the system’s control loops in terms of stability. In addition, it gives guidelines to assess the stability, which may be extended to other topologies where frequency variation is expected. In particular, the main contribution of this paper is a systematic procedure to quantify the stability range of well-known *dq* SRF-based control schemes that use *static* and *dynamic decouplers* such as [35,36,38]. Theoretical and experimental results are included in a proof-of-concept prototype in order to validate the proposed analysis.

The analysis first considers the study of a SRF-based linear controller with a *static decoupler* [39]. Then, a second one is analyzed, this time based on a *dynamic decoupler*, which explicitly includes the time-variant feature of the ac mains frequency. An analysis is performed for the *static* and *dynamic decoupler* through the Lyapunov stability criteria to find the eigenvalues considering a micro-grid with variable frequency and, thus, conclude stability issues under the aforementioned considerations.

The paper is organized as follows. Section 2 presents the model of the power converter under variable grid frequency. Section 3 presents the control algorithms with the decouplers. Section 4 shows the theoretical stability analysis for the controllers. Section 5 shows simulated results which illustrate the issues. Section 6 shows experimental results which validate the theoretical analysis. Section 7 discusses the results. Finally, Section 8 indicates the main conclusions from this study.

## 2. Modeling of Power Converters under Variable Grid Frequency

**Figure 1:**
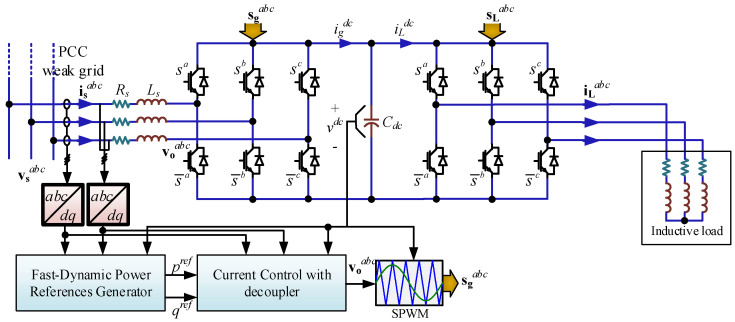
Power Converter System.

The grid-connected power converter can be modeled by explicitly considering a variable grid frequency [4,22]. The rectifier side of the system shown in Figure 1 is modeled through the Kirchhoff’s current and voltage laws, resulting in

(1) $$C_{dc}\frac{dv^{dc}}{dt}=i_{g}{}^{dc}-i_{L}{}^{dc}=G_{ac}\left({\left(m_{g}{}^{abc}\right)}^{T}i_{g}{}^{abc}-{\left(m_{L}{}^{abc}\right)}^{T}i_{L}{}^{abc}\right)$$

(2) $$v_{g}{}^{abc}=L_{g}\frac{di_{g}{}^{abc}}{dt}+R_{g}i_{g}{}^{abc}+G_{ac}m_{g}{}^{abc}v^{dc}$$

where *G_ac_* is the modulation technique gain (*G_ac_* = 0.866 for plain sinusoidal PWM), **m_g_***^abc^* and **m_L_***^abc^* are the rectifier and the inverter modulating signals, respectively.

Equations (1) and (2) can be rewritten in the rotating reference frame by the use of the Park Transformation, leading to the following state variable equations:

(3) $$\frac{dv^{dc}}{dt}=\frac{G_{ac}}{C_{dc}}\left(m_{g}{}^{d}i_{g}{}^{d}+m_{g}{}^{q}i_{g}{}^{q}\right)-\frac{1}{C_{dc}}i_{L}^{dc}$$

(4) $$\frac{di_{g}{}^{d}}{dt}=\frac{-R_{g}}{L_{g}}i_{g}{}^{d}+\omega_{g}i_{g}{}^{q}-\frac{1}{L_{g}}G_{ac}m_{g}{}^{d}v^{dc}+\frac{1}{L_{g}}v_{g}{}^{d}$$

(5) $$\frac{di_{g}{}^{q}}{dt}=\frac{-R_{g}}{L_{g}}i_{g}{}^{q}-\omega_{g}i_{g}{}^{d}-\frac{1}{L_{g}}G_{ac}m_{g}{}^{q}v^{dc}+\frac{1}{L_{g}}v_{g}{}^{q}$$

where ω*_g_* is the variable grid frequency in rad/s. The model shows a new nonlinearity due to the frequency ω*_g_*, which is considered as a disturbance on the currents *i_g_^d^* and *i_g_^q^* in (4) and (5), respectively. This nonlinearity requires a careful choice of control technique in order to obtain proper dynamic and static behavior.

In order to simplify the mathematical analysis, the state function **f** is defined after writing (3)–(5) in the standard nonlinear system form as

(6) dx/dt=f(x,u,p)

where **x** = [*v^dc^ i_g_^d^ i_g_^q^*]*^T^*, **u** = [*m_g_^d^ m_g_^q^*]*^T^*, **p** = [*v_g_^d^ v_g_^q^* ω*_g_ i_L_^dc^*]*^T^* for the rectifier control side. The disturbances are the grid voltage and frequency, and the *dc* current of the inverter side. A more detailed analysis of the system model can be found in [38].

## 3. Control of Power Converters under Variable Grid Frequency

The system in Figure 1 shows a variable frequency voltage source, but a constant frequency is desired at the load side. This topology can be found on micro-grids where the frequency may deviate from the operating point, but the load requires a fixed frequency.

### 3.1. Control Goals

Two control techniques are used to operate the system in Figure 1, when a variable frequency environment is considered: (i) SRF linear controller with a *static decoupler*, and (ii) SRF linear controller with *dynamic decoupler* based on state variables and disturbances feed-forward. Both controllers have the same objectives, which are to control the active power (to regulate the *dc* voltage) and the reactive power (to regulate the grid power factor). The *dc* link voltage can be controlled by the direct axis current and the power factor through the quadrature current.

### 3.2. Linear Control with Static Decoupler

The first case of study uses linear controllers with a *static decoupler*, which is designed for a specific operating point. To obtain the *static decoupler*, the linear model must be found from (6) [33], given by:

(7) $$\Delta \dot{x}=A\Delta x+B\Delta u+E\Delta p$$

with:

(8) $$A=\frac{df\left(x_{o},u_{o},p_{o}\right)}{dx};B=\frac{df\left(x_{o},u_{o},p_{o}\right)}{du};E=\frac{df\left(x_{o},u_{o},p_{o}\right)}{dp}$$

the linear system matrices, and Δ**x** = **x** − **x_o_**, Δ**u** = **u** − **u_o_**, Δ**p** = **p** − **p_o_**, the state variables. The inputs and the disturbances associated with a chosen operating point are described by the subscript ‘**_o_**’.

From the current control point of view, *i_g_^d^* and *i_g_^q^* are the variables of interest. Hence, the output vector, namely **y**, can be written as:

(9) $$\Delta y=C\Delta x=\left(\begin{array}{ccc} 0 & 1 & 0\\ 0 & 0 & 1 \end{array}\right)\left(\begin{array}{c} v_{dc}-v_{dc}{}_{o}\\ i_{g}^{d}-i_{g}^{d}{}_{o}\\ i_{g}^{q}-i_{g}^{q}{}_{o} \end{array}\right)$$

From (8) and (9), the Matrix Transfer Function, which gives the linear representation [40], becomes:

(10) $$H\left(s\right)=C{\left(sI-A\right)}^{-1}B$$

and the *static decoupler* [33] is:

(11) $$\begin{array}{l} H^{-1}\left(0\right)=-{\left(CA^{-1}B\right)}^{-1}\\ \;\;\;\;\;\;\;\;\;=\frac{\left(\begin{array}{cc} {\left(m_{g}^{d}{}_{o}\right)}^{2}v_{dc}{}_{o}+\alpha_{1} & m_{g}^{d}{}_{o}m_{g}^{q}{}_{o}v_{dc}{}_{o}+\alpha_{2}\\ m_{g}^{d}{}_{o}m_{g}^{q}{}_{o}v_{dc}{}_{o}+\alpha_{3} & {\left(m_{g}^{q}{}_{o}\right)}^{2}v_{dc}{}_{o}+\alpha_{4} \end{array}\right)}{G_{ac}\left(i_{g}^{d}{}_{o}m_{g}^{d}{}_{o}+i_{g}^{q}{}_{o}m_{g}^{q}{}_{o}\right)v_{dc}{}_{o}} \end{array}$$

where:

$$\alpha_{1}=-L_{g}i_{g}^{q}{}_{o}m_{g}^{d}{}_{o}\omega_{g}{}_{o}+R_{g}i_{g}^{q}{}_{o}m_{g}^{q}{}_{o},\;\alpha_{2}=-L_{g}i_{g}^{q}{}_{o}m_{g}^{q}{}_{o}\omega_{g}{}_{o}-R_{g}i_{g}^{q}{}_{o}m_{g}^{d}{}_{o},\;\alpha_{3}=L_{g}i_{g}^{d}{}_{o}m_{g}^{d}{}_{o}\omega_{g}{}_{o}-R_{g}i_{g}^{d}{}_{o}m_{g}^{q}{}_{o},\;\alpha_{4}=L_{g}i_{g}^{d}{}_{o}m_{g}^{q}{}_{o}\omega_{g}{}_{o}+R_{g}i_{g}^{d}{}_{o}m_{g}^{d}{}_{o}.$$

Note that all the quantities in **H**^−1^(0) are chosen for a specific operating point.

**Figure 2:**
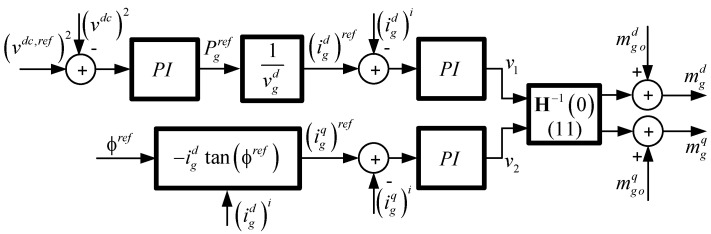
Power converter rectifier with a static decoupler control.

The *static decoupler* (11), designed for a certain operating point, provides a decoupled current control in steady state. Therefore, in order to achieve zero steady state error, two independent Proportional-Integral (PI) controllers are used with the form:

(12) $$h_{c}\left(s\right)=k_{p}^{i_{g}}\left(1+\frac{1}{T_{i}^{i_{g}}s}\right)$$

where *k_p_* is the proportional gain and *T_i_* is the integrative time. These PI controllers are also designed for a specific operating point to obtain a desired dynamic and static performance for a fixed frequency *ω_go_* [33,34]. The control system scheme is shown in Figure 2, including the *dc* voltage and the power factor control loops.

### 3.3. Linear Control with Dynamic Decoupler

A *dynamic decoupler* is another option to linearize and decouple the system [33]. Similar to the *static decoupler* case, the *dc* voltage control is a master loop on the direct current, while the required power factor gives the quadrature current reference.

The *dynamic decoupler* is found deriving the equation that defines the output variables and making them equal to new input variables that herein are named *w*_1_ and *w*_2_:

(13) $$\frac{di_{g}^{d}}{dt}=w_{1},\frac{di_{g}^{q}}{dt}=w_{2}$$

where the left side in the last equations are (4) and (5). Thus, from (4), (5) and (13), it is found:

(14) $$\frac{di_{g}^{d}}{dt}=\frac{-R_{g}}{L_{g}}i_{g}{}^{d}+\omega_{g}i_{g}{}^{q}-\frac{1}{L_{g}}G_{ac}m_{g}{}^{d}v^{dc}+\frac{1}{L_{g}}v_{g}{}^{d}=w_{1}$$

(15) $$\frac{di_{g}^{q}}{dt}=\frac{-R_{g}}{L_{g}}i_{g}{}^{q}-\omega_{g}i_{g}{}^{d}-\frac{1}{L_{g}}G_{ac}m_{g}{}^{q}v^{dc}+\frac{1}{L_{g}}v_{g}{}^{q}=w_{2}$$

and solving for *m_g_^d^* and *m_g_^q^*, the resulting *dynamic decoupler* to be implemented is:

(16) $$m_{g}^{d}={\left(G_{ac}v^{dc}\right)}^{-1}\left\{-L_{g}w_{1}-R_{g}{\left(i_{g}^{d}\right)}^{i}+L_{g}\omega_{g}^{i}{\left(i_{g}^{q}\right)}^{i}+{\left(v_{g}^{d}\right)}^{i}\right\}$$

(17) $$m_{g}^{q}={\left(G_{ac}v^{dc}\right)}^{-1}\left\{-L_{g}w_{2}-R_{g}{\left(i_{g}^{q}\right)}^{i}-L_{g}\omega_{g}^{i}{\left(i_{g}^{d}\right)}^{i}+{\left(v_{g}^{q}\right)}^{i}\right\}$$

where the superscript *i* means that the variable is obtained from the Park Transformation, which uses the frequency estimated by the PLL and may be different from the real variable. Indeed, the Park Transformation angular position θ*^i^* given by the PLL is an estimation of the real angle position θ.

With the *dynamic decoupler* defined in (16) and (17), the system defined by the current *i_g_*^{*d*,*q*}^ and the input *w*_{1,2}_ behaves as an integrator. In order to obtain a first order linear response (*h*(*s*) = *k_p_*/(τ*s* + 1) = *i_g_*^{*d,q*}^/*v*_{1,2}_), the inputs *w*_1_ and *w*_2_ are redefined as:

(18) $$w_{1}=\left(1/\tau \right){\left(i_{g}^{d}\right)}^{i}+k_{p}\left(1/\tau \right)v_{1}$$

(19) $$w_{2}=\left(1/\tau \right){\left(i_{g}^{q}\right)}^{i}+k_{p}\left(1/\tau \right)v_{2}$$

where **v** = [*v*_1_ *v*_2_]*^T^* is the new input vector to impose a first order response. Finally, the modulation signals are:

(20) $$m_{g}^{d}=\frac{-\frac{L_{g}}{\tau }\left(-{\left(i_{g}^{d}\right)}^{i}+k_{p}v_{1}\right)-R_{g}{\left(i_{g}^{d}\right)}^{i}+L_{g}\omega_{g}^{i}{\left(i_{g}^{q}\right)}^{i}+{\left(v_{g}^{d}\right)}^{i}}{G_{ac}v^{dc}}$$

(21) $$m_{g}^{q}=\frac{-\frac{L_{g}}{\tau }\left(-{\left(i_{g}^{q}\right)}^{i}+k_{p}v_{2}\right)-R_{g}{\left(i_{g}^{q}\right)}^{i}-L_{g}\omega_{g}^{i}{\left(i_{g}^{d}\right)}^{i}+{\left(v_{g}^{q}\right)}^{i}}{G_{ac}v^{dc}}$$

Equations (20) and (21) show $\omega_{g}^{i}$ as a necessary variable to implement the *dynamic decoupler*, with $\omega_{g}^{i}$ being the estimated angular frequency of the *ac* mains. This frequency is obtained from the PLL [5,22], as:

(22) $$\omega_{g}^{i}=2\pi /\left(NT_{s}\right)$$

Note that the PLL ensures $\omega_{g}^{i}$ equal to $\omega_{g}$ in a steady state and it is a good approximation during transient conditions [1,2,3,4,5,6,7,8,9,10,11,12,13,14]. Moreover, in this work, the *ac* mains frequency is estimated at every sampling time by means of a discrete three-phase SRF PLL. This approach also guarantees a fixed number *N* of samples per period, as the sampling time *T_s_* is also adjusted [3,5].

The SRF variables in (20) and (21), which are identified by the superscript *i*, also denotes the *dq* variables obtained from the Park Transformation. The relationship between the real *x* (voltage or current) and the estimated *x^i^* is:

(23) $${\left(x^{d}\right)}^{i}=\sqrt{{\left(x^{d}\right)}^{2}+{\left(x^{q}\right)}^{2}}\cos\left(\theta^{i}-\theta +\tan^{-1}\left(\frac{x^{q}}{x^{d}}\right)\right)$$

(24) $${\left(x^{q}\right)}^{i}=\sqrt{{\left(x^{d}\right)}^{2}+{\left(x^{q}\right)}^{2}}\sin\left(\theta^{i}-\theta +\tan^{-1}\left(\frac{x^{q}}{x^{d}}\right)\right)$$

Naturally, if θ*^i^* = θ, i.e., the PLL is in a steady state, then (*x^d^*)*^i^* = *x^d^* and (*x^q^*)*^i^* = *x^q^*. It is important to highlight that the difference between the real and the estimated variable is small, since PLLs are now very accurate [1,2,3,4,5,6,7,8,9,10,11,12,13,14]. This justifies assuming both variables are identical for control design and analysis.

**Figure 3:**
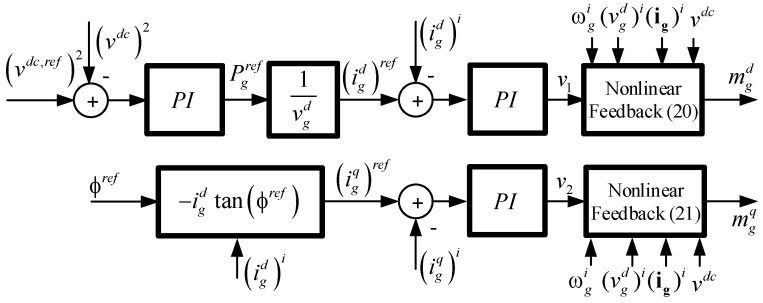
Power converter rectifier with a dynamic decoupler control.

Finally, a similar control structure of the *static decoupler*, Figure 2, is applied to the designed *dynamic decoupler* as shown in Figure 3, controlling both the *dc* link voltage and the power factor by means of a PI controller. For discrete control implementation, the PI controllers of Figure 2 and Figure 3 must be mapped to the Z-plane, as explained in [37,38].

## 4. Stability Analysis Procedure

An important issue about the previous controllers is their stability within a certain range of frequency and/or their operation in the presence of frequency deviations for applications such as micro-grids and weak grids, among others. The open loop model (6) is a nonlinear and coupled system, therefore the Lyapunov stability criteria can be used to study the effect of frequency variations.

### 4.1. Stability Analysis of Static Decoupler Control

The *static decoupler* is computed for a nominal operating point, but this condition, especially the frequency, could shift away from the original value and lead to an unstable control system. This is expected because the decoupler depends on the state variables, inputs, disturbances, and parameters (11), besides the frequency at the operating point. In order to analyze the system response under grid frequency variations, a stability study is performed in this section for the global closed loop system shown in Figure 2.

The modulating signals, depending upon the current controller outputs and the *static decoupler*, can be written as:

(25) $$\left(\begin{array}{c} \Delta m_{g}^{d}\\ \Delta m_{g}^{q} \end{array}\right)=H^{-1}\left(0\right)\left(\begin{array}{c} v_{1}\\ v_{2} \end{array}\right)=K\left(\begin{array}{c} v_{1}\\ v_{2} \end{array}\right)=\left(\begin{array}{cc} k_{11} & k_{12}\\ k_{21} & k_{22} \end{array}\right)\left(\begin{array}{c} v_{1}\\ v_{2} \end{array}\right)$$

where $\Delta m_{g}^{\left\{d,q\right\}}=m_{g}^{\left\{d,q\right\}}-m_{g}^{\left\{d,q\right\}}{}_{o}$.

As it was stated in Section 4, the quadrature current reference is given by the desired input power factor, and the direct current is given by the *dc* voltage control output. Because both current control loops use a PI controller (12), the current control state variables representation of (12) can be written as:

(26) $${\dot{\zeta }}_{1}=a_{c}\zeta_{1}+b_{c}\left({\left(i_{g}^{d}\right)}^{ref}-i_{g}^{d}\right),\;v_{1}=c_{c}\zeta_{1}+d_{c}\left({\left(i_{g}^{d}\right)}^{ref}-i_{g}^{d}\right)$$

(27) $${\dot{\zeta }}_{2}=a_{c}\zeta_{2}+b_{c}\left({\left(i_{g}^{q}\right)}^{ref}-i_{g}^{q}\right),v_{2}=c_{c}\zeta_{2}+d_{c}\left({\left(i_{g}^{q}\right)}^{ref}-i_{g}^{q}\right)$$

where *a_c_* = 0, *b_c_* = 1, *c_c_* = *k_c_*/*T_i_*, *d_c_* = *k_c_* are the current controller parameters. On the other hand, the PI for the *dc* voltage control can be expressed as:

(28) $$\begin{array}{l} {\dot{\zeta }}_{3}=a_{c}^{v^{dc}}\zeta_{3}+b_{c}^{v^{dc}}\left({\left(v_{}^{dc,ref}\right)}^{2}-{\left(v^{dc}\right)}^{2}\right),\\ {\left(i_{g}^{d}\right)}^{ref}=c_{c}^{v^{dc}}\zeta_{3}+d_{c}^{v^{dc}}\left({\left(v_{}^{dc,ref}\right)}^{2}-{\left(v^{dc}\right)}^{2}\right) \end{array}$$

where *a_c_^vdc^* = 0, *b_c_^vdc^* = 1, *c_c_^vdc^* = *k_c_*/*T_i_*, *d_c_^vdc^* = *k_c_* are the voltage controller parameters.

Using the state variables representation of the current controller (26) and (27) and the *dc* voltage controller (28), the overall closed loop system model can be written in the state variable form. This is defined by the union (closed loop, Figure 2) of the open loop model (3)–(5), the *static decoupler* (25), the current controller (26) and (27) and the *dc* voltage controller (28) in one equation, leading to the following nonlinear state variable representation [38,41]:

(29) $$\frac{dx_{L}}{dt}=f_{L}\left(x_{L},u_{L},p_{L}\right)$$

where $x_{L}={\left[v^{dc},\;i_{g}^{d},\;i_{g}^{q},\;\zeta_{1},\;\zeta_{2},\;\zeta_{3}\right]}^{T}$, and $u_{L}={\left[{\left(v_{}^{dc,ref}\right)}^{2},\;{\left(i_{g}^{q}\right)}^{ref}\right]}^{T}$. The stability of the resulting system (29) can be studied applying Lyapunov’s method, which verifies the eigenvalues in each operating point of the Jacobian matrix:

(30) $$A_{L}=\frac{\partial f_{L}\left(x_{L},u_{L},p_{L}\right)}{\partial x_{L}}$$

**Figure 4:**
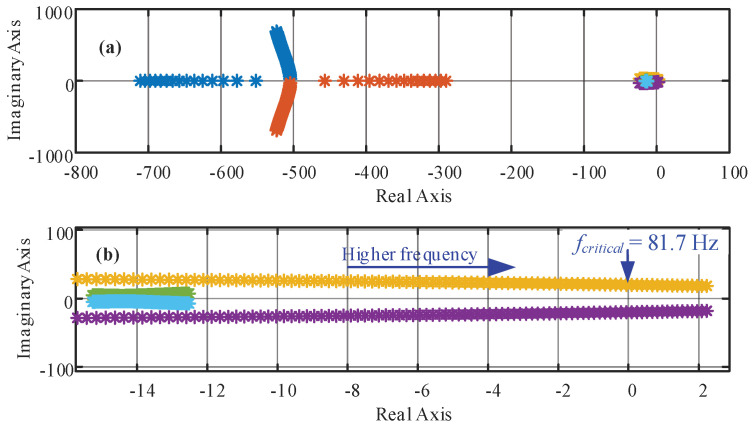
**A_L_** eigenvalues as a function of the *ac* main frequency (from 30 to 100 Hz); (**a**) all six values; (**b**) zoom to the unstable eigenvalues.

**Figure 5:**
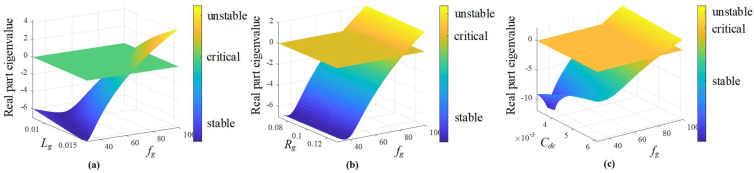
The imaginary closest eigenvalue evolution as a function of parameters with frequency (from 30 to 100 Hz). (**a**) *L_g_* from 70% to 130% with respect the nominal value; (**b**) *R_g_* from 70% to 130% with respect the nominal value; (**c**) *C_dc_* from 70% to 130% with respect the nominal value.

The passive filtering components utilized in the topology, Figure 1, are designed considering the switching frequency and further details are given in [40]. For the eigenvalues study, a power factor at the source of *pf_g_* = 0.93(*i*) and a *dc* voltage *v^dc^* = 750 (V) with grid frequencies from 30 (Hz) to 100 (Hz) are imposed. Figure 4 shows the eigenvalues with the parameters given in Table 1. Figure 4b highlights the eigenvalues nearest to the imaginary axis, where the critical grid frequency is 81.7 Hz. It is worth noting that the value of the critical frequency is strongly dependent on the parameters and the operating point, as shown in Table 2 in percentage with respect the nominal value. However, the most variation in the critical frequency is related to the inductance value, and, on the contrary, the losses in the inductances (the resistance *R_g_*) are the ones that less affect the stable zone. This is highlighted in Figure 5, where Figure 5a shows the response for *L_g_*, where the higher the value of *L_g_* is, the lower the critical frequency is (where the eigenvalues are equal to zero). On the other hand, Figure 5b,c shows critical frequency little dependence with respect to *R_g_* and *C_dc_*.

As aforementioned, high frequencies lead some eigenvalues to reach positive real values, making the closed loop system unstable, as confirmed in the next section. The eigenvalues that become unstable for high frequencies are the ones given by the first order *L*-type input filter that are defined by the impedance *R_g_* + *j*ω*_g_L_g_*. These eigenvalues are already very close to the imaginary axis in open loop because *R_g_* → 0, where *R_g_* represents the resistive losses of the filter. Moreover, the imaginary part is related to the *ac* mains frequency.

### 4.2. Stability Analysis of Dynamic Decoupler Control

The error introduced by the PLL was not considered in the stability analysis of the linear control with a *static decoupler* because the control strategy is unstable even when an ideal PLL is considered. On the other hand, the error introduced by the PLL in the linear control with a *dynamic decoupler* needs to be considered because the nonlinear feedback uses the estimated frequency, affecting the overall performance.

The analysis was carried out in the continuous domain to reduce the mathematical burden, thus, the PLL is considered as a second order transfer function, *h_PLL_*(*s*) = ω*_n_*^2^/(*s*^2^ + 2ξω*_n_s* + ω*_n_*^2^), which should represent as close as possible the PLL response. Hence, the PLL state variables representation can be written as:

(31) $$\frac{d\delta_{1}}{dt}=\delta_{2}$$

(32) $$\frac{d\delta_{2}}{dt}=-\omega_{n}{}^{2}\delta_{1}-2\xi \omega_{n}\delta_{2}+\omega_{n}{}^{2}\frac{1}{N\cdot f_{g}}$$

where ω*_n_* is the PLL natural frequency and ξ is the PLL damping factor. The variable *f_g_* is the real grid frequency and δ_1_ = *T_s_*, where the estimated frequency can be found as (22).

To study the global system stability, the *dynamic decoupler* must be included, which leads to applying the *dynamic decoupler* (20) and (21) in the system model given by (3)–(5), resulting in (33)–(35):

(33) $$\frac{di_{g}{}^{d}}{dt}=\frac{-1}{\tau }{\left(i_{g}^{d}\right)}^{i}+\frac{k_{p}}{\tau }v_{1}+\frac{-R_{g}}{L_{g}}\left(i_{g}^{d}-{\left(i_{g}^{d}\right)}^{i}\right)+\left(\omega_{g}i_{g}^{q}-\frac{{\left(i_{g}^{q}\right)}^{i}}{NT_{s}}\right)+\frac{1}{L_{g}}\left(v_{g}^{d}-{\left(v_{g}^{d}\right)}^{i}\right)$$

(34) $$\frac{di_{g}{}^{q}}{dt}=\frac{-1}{\tau }{\left(i_{g}^{q}\right)}^{i}+\frac{k_{p}}{\tau }v_{2}+\frac{-R_{g}}{L_{g}}\left(i_{g}^{q}-{\left(i_{g}^{q}\right)}^{i}\right)-\left(\omega_{g}i_{g}^{q}-\frac{2\pi {\left(i_{g}^{q}\right)}^{i}}{NT_{s}}\right)+\frac{1}{L_{g}}\left(v_{g}^{q}-{\left(v_{g}^{q}\right)}^{i}\right)$$

(35) $$\frac{dv^{dc}}{dt}=\frac{G_{ac}}{C_{dc}}\left(\begin{array}{l} \frac{1}{G_{ac}v^{dc}}\left\{\begin{array}{l} -L_{g}\left(\left(-1/\tau \right){\left(i_{g}^{d}\right)}^{i}+\left(k_{p}/\tau \right)v_{1}\right)-R_{g}{\left(i_{g}^{d}\right)}^{i}+\cdots \\ L_{g}\left(2\pi /\left(NT_{s}\right)\right){\left(i_{g}^{q}\right)}^{i}+{\left(v_{g}^{d}\right)}^{i} \end{array}\right\}i_{g}^{d}+\cdots \\ \frac{1}{G_{ac}v^{dc}}\left\{\begin{array}{l} -L_{g}\left(-1/\tau \right){\left(i_{g}^{q}\right)}^{i}+\left(k_{p}/\tau \right)v_{2}-R_{g}i{\left(i_{g}^{q}\right)}^{i}+\cdots \\ -L_{g}\left(2\pi /\left(NT_{s}\right)\right){\left(i_{g}^{d}\right)}^{i}+{\left(v_{g}^{q}\right)}^{i} \end{array}\right\}i_{g}^{q} \end{array}\right)-\frac{1}{C_{dc}}i_{L}{}^{dc}$$

At this point, the effect of the sampling time *T_s_* shows up, because if $\omega_{g}=2\pi /\left(NT_{s}\right)$ (when the PLL is perfectly synchronized), all parenthesis in (33) and (34) add up to zero, and the *dynamic decoupler* obtains the desired time constant τ between *i_g_* and *v* (just the two first terms).

Then, including the state variables related to the PLL (31) and (32), the open loop system (3)–(5) and the PI controllers (26)–(28), the state variable representation for the overall closed loop system with nonlinear control is summarized in (36),

(36) $$dx_{NL}/dt=f_{NL}\left(x_{NL},u_{NL},p_{NL}\right)$$

with $x_{NL}^{T}=[\delta_{1},\delta_{2},v_{}^{dc},\;i_{g}^{d},\;i_{g}^{q},\;\zeta_{1},\;\zeta_{2},\;\zeta_{3}]$, $p_{NL}^{T}=[v_{g}^{d},\;v_{g}^{q},\;f_{g},\;i_{L}^{dc}]$, $u_{NL}^{T}=[{\left(v_{}^{dc,ref}\right)}^{2},{\left(i_{g}^{q}\right)}^{ref}]$, and is shown in Figure 3.

The closed loop stability study is performed through Lyapunov’s method, finding the Jacobian matrix from (36) as:

(37) $$A_{NL}=\frac{\partial f_{NL}\left(x_{NL},u_{NL},p_{NL}\right)}{\partial x_{NL}}$$

**Figure 6:**
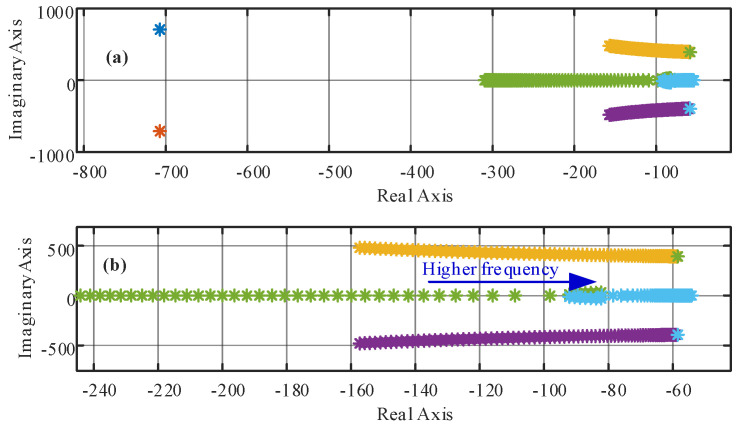
**A_NL_** eigenvalues as a function of the *ac* main frequency (from 30 to 100 Hz), (**a**) all six values, (**b**) zoom to the unstable eigenvalues.

If it is assumed that the PLL provides the exact grid frequency at all times, then the Jacobian eigenvalues in (37) are only constant values due to the *dynamic decoupler*. The *dynamic decoupler* linearizes and decouples the system in the entire operating region and fixes the eigenvalues in a desired position [38]. However, if the PLL does not provide the exact *ac* mains frequency, the *dynamic decoupler* ends up using an approximated *ac* mains frequency. This frequency deviation implies that the summation associated with the frequency terms in (33) and (34) would be different to zero and the dynamic between *i_g_* and *v* will not be a first order type. This could happen when the PLL has an error in the sensed variables, when the synchronization fails or even under bad scaling of the sensed variables. In this case, as the two first terms in (33) and (34) may not be equal at all times, the current dynamic is not the imposed in (18) and (19), and the eigenvalues may go away from their desired position. To illustrate this effect, Figure 6 shows the eigenvalues evolution, with the parameters given in Table 1, when the PLL frequency presents a deviation from the grid frequency. The variation tested considers an actual frequency of 50 (Hz), while the estimated one goes from 30 (Hz) up to 100 (Hz). Even under this big deviation, the eigenvalues of the system are stable and distant from the instability zone, showing the robustness of the dynamic decoupling stage under varying frequency conditions.

## 5. Simulated Results

To verify the previous mathematical analysis, the system of Figure 1 is simulated with the parameters listed in Table 1. First, a power factor change from *pf* = 0.93(*i*) to *pf* = 1.00(*i*) in *t* = 0.1 (s) is imposed. Then, a frequency change is applied from *f_g_* = 50 (Hz) to *f_g_* = 30 (Hz) in *t* = 0.15 (s) and to *f_g_* = 90 (Hz) (*t* > 0.5 s). Finally, a *dc* voltage reference change is carried out in *t* = 0.4 (s) from *v^dc^* = 750 (V) to *v^dc^* = 800 (V).

### 5.1. Static Control Response

**Figure 7:**
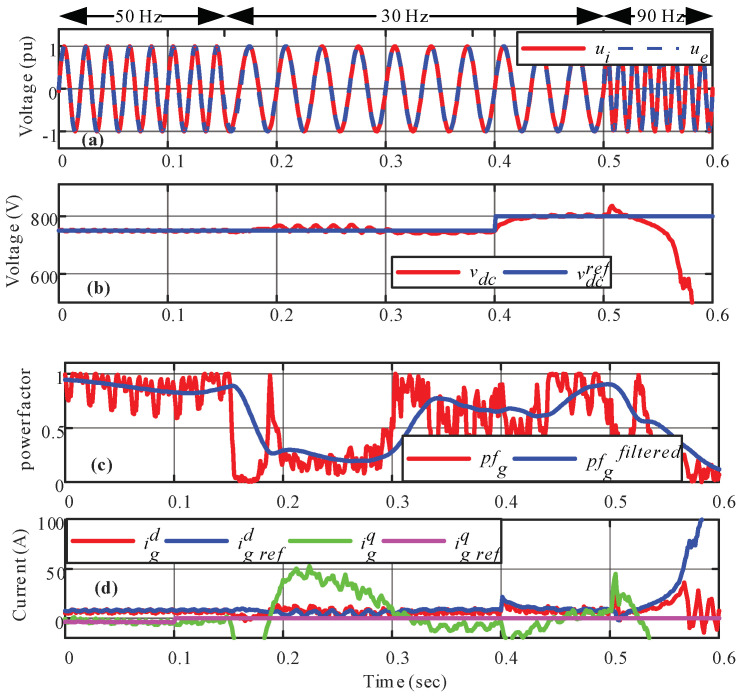
Linear control response; (**a**) synchronization; (**b**) *dc* voltage; (**c**) power factor; (**d**) input rectifier currents.

In Figure 7a, a satisfactory synchronization between external signal and internal signal can be seen, which shows the correct tracking of the PLL. After *t* = 0.5 (s), when the grid frequency is changed to 80 (Hz), the controller fails to regulate the *dc* link voltage as shown in Figure 7b. It is evident that the controller performance is not appropriate when the grid frequency changes. The currents dynamic behavior is clearly affected, Figure 7d, from *t* = 0.15 (s). When the grid frequency goes down before *t* = 0.5 (s), the *L*-type input filter changes its impedance (given by *R_g_* + *j*ω_g_*L*_g_) which produces a transient in the input current, which is compensated by the closed loop. However, because the *static decoupler* was designed ensuring stability for a specific operating point at 50 (Hz), the system becomes unstable in *t* = 0.5 (s) when the frequency increases to 80 (Hz), confirming the results of Section 5. On the other hand, Figure 7c shows the power factor, which exhibits important oscillations, as the *static decoupler* was designed for a different operating point, anticipating possible damage to the converter and the disconnection of the load.

### 5.2. Dynamic Control Response

**Figure 8:**
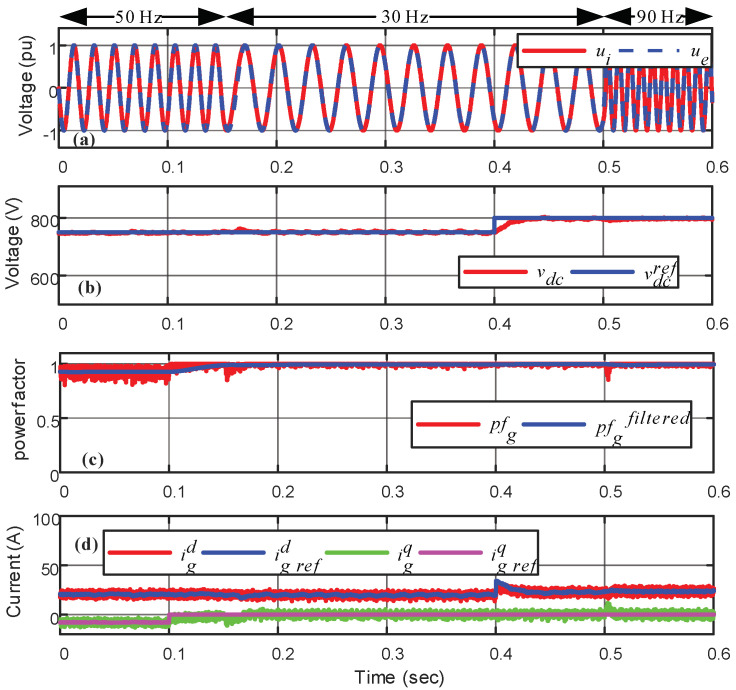
Nonlinear control response; (**a**) synchronization; (**b**) *dc* voltage; (**c**) power factor; (**d**) input rectifier currents.

Figure 8 shows the response of the controller that uses the *dynamic decoupling* scheme. Compared to the *static decoupler* control, it has satisfactory dynamic and static responses. The *dc* voltage and power factor are well controlled and follow their references, while the internal current control loops operate without significant oscillations under the safe operation of the converter and load. As the estimated grid frequency is fed-forward into the controller, this strategy results in more robust frequency variations.

## 6. Experimental Results

**Figure 9:**
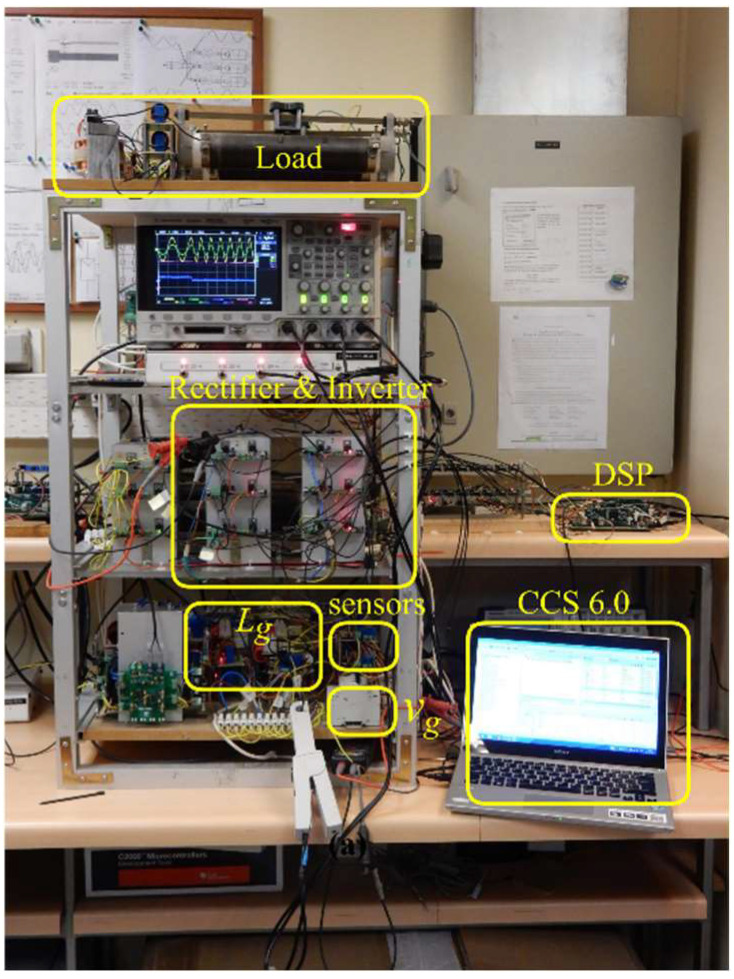
Experimental three-phase grid-connected power converter system.

In order to corroborate the theoretical and simulated results, a proof-of-concept prototype of the power converter shown in Figure 1 is implemented. The dynamic decoupling control algorithm was programmed in a TMS320F28335 DSP-based board. The complete experimental setup is presented in Figure 9.

**Figure 10:**
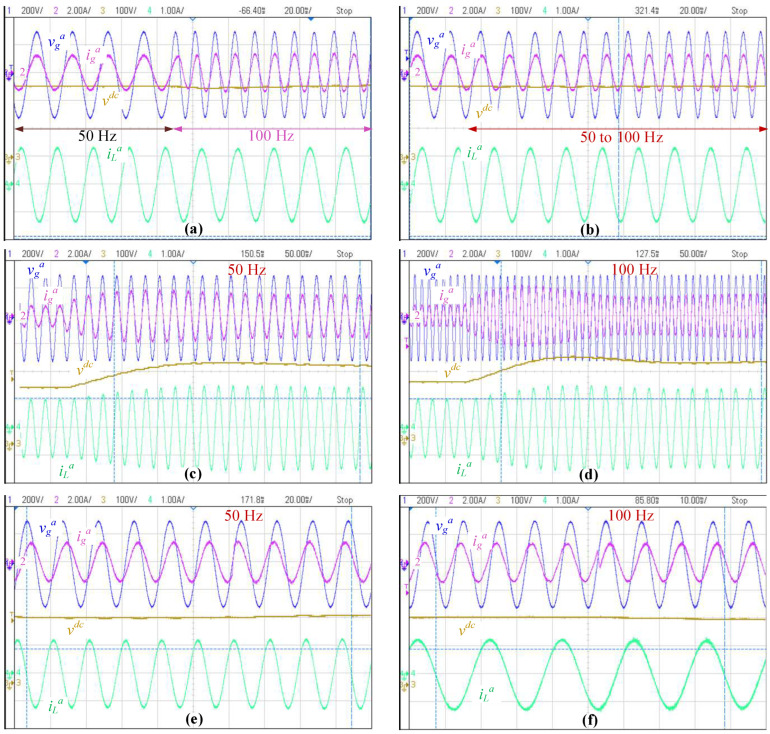
Results for dynamic decoupler; (**a**) step-up frequency change; (**b**) ramp-up frequency change; (**c**) *dc* voltage step-up response with *f_g_* = 50 Hz; (**d**) *dc* voltage step-up response with *f_g_* = 100 Hz; (**e**) power factor step response with *f_g_* = 50 Hz; (**f**) power factor step response with *f_g_* = 100 Hz.

Naturally, all the changes in real applications are smooth or ramp-type with a given duration. However, the mainly employed input for control purposes is the step, because it makes it possible to find several important parameters in the response of linear systems as overshoot, settling band and time. In this context, a step in the frequency is performed from 50 Hz to 100 Hz, which can be seen in Figure 10a. Although this process is nonlinear and despite the huge frequency change, it can be seen in Figure 10a that the control remains stable and after coupler cycles, the power factor becomes unitary and the *dc* voltage returns to its previous value, even though the *dc* voltage is affected only a little. To complement the tests, Figure 10b shows a ramp-up frequency sweep, where in this case, the power factor and the voltage remain very close to their references and no ramble can be perceived, displaying the good performance under frequency variation of the dynamic decoupler.

A *dc* voltage step-up change is performed and depicted in Figure 10c,d for *f_g_* = 50 and 100 (Hz), respectively. As can be seen, the dynamic decoupler-based controller is capable of working in this wide variation frequency, as expected and theoretically demonstrated by the stability analysis. On the other hand, both responses look to be similar, even when the frequency—and, as a consequence, the impedance—changes by 100%.

The power factor response is tested and the results are presented in Figure 10e,f. The power factor goes from *fp* = 0.8 inductive to *fp* = 0.8 capacitive. The results show a similar behavior for *f_g_* =50 Hz in Figure 10e and when *f_g_* = 100 (Hz) in Figure 10f, even the wide frequency range tested. As stated previously for the *dc* voltage control, the frequency does not affect much in the dynamic behavior, and again, the control remains stable, as expected and demonstrated mathematically.

Since the tests using the *static decoupler* are destructive, as presented in Figure 7, the simulations performed in the previous section are used as a basis for comparison, which show instabilities below the 80 Hz, Table 2. Although the *dynamic decoupler* has a better behavior, its computation effort is greater than the *static decoupler*, and a dedicated digital board is required. Nevertheless, it is important to highlight that the computing time is similar for both cases because most of the time is required for the sensed variables acquisition, PI controllers, PLLs and the rest of the DSP related functions.

## 7. Discussion

The behavior of two *dq* SRF based control schemes based on static and dynamic decouplers has been studied and tested in the presence of wide frequency variations. The theoretical and simulation results show that under frequency variations, the system could reach instability depending upon the chosen control approach, particularly in the case when a *static decoupler* is considered, which is the most simple and common approach. Although the controller based on this approach remains stable up to 80 Hz for the used parameter set, oscillations with big excursions are obtained. Additionally, if the system’s parameters vary; for instance, the filter inductor, the stability region for the frequency can be substantially reduced, leading to a 25% reduction for a 30% increase in the inductor value. On the other hand, the dynamic decoupler-based controller shows a larger stability region than the static one. Indeed, an increase of up to 100% in the frequency does not induce instability. It can be concluded from the results that from the control point of view, although *dynamic decouplers* are computationally heavier, in some cases they are required to ensure proper behavior. A comparison of key characteristics of both approaches is summarized in Table 3.

The proposed procedure based on Lyapunov criteria can be extended to any other grid—connected power converter configuration—as a current source or voltage source converters in order to verify the closed loop system’s stability. Furthermore, this study can be extended to any *dynamic decoupler* [34,36,38] and *static decoupler* [33]-based controller, and topologies with LCL filters where four new state variables would be added (6). The procedure can be summarized as follows:

➢ Define the model in the variable state representation;
➢ Define the controllers in the variable state representation;
➢ Build the representation of the whole system, including all the dynamics in the power converters, as currents, voltages, PLL internal dynamics, controllers, etc.;
➢ Determine the Jacobian as a function of the rest of the variables;
➢ Find the eigenvalues as a function of the variables of interest such as frequency, parameters and operating points where the system would work.

The aforementioned procedure can give useful guidelines, making it possible to characterize the operation of power converters controllers under frequency variations.

## 8. Conclusions

This work has comprehensively analyzed the stability characteristics and behavior of SRF-based linear-based controllers as used in grid-connected three-phase power converters when they are connected to a variable supply frequency environment. These converters, which are used as key building blocks of renewable energy interfaces and motor drives, are likely to operate under these conditions if used in micro and weak grids, where important frequency variations are expected. The theoretical analysis based on the Lyapunov indirect method has considered a static decoupler-based SRF controller, which effectively considers the system’s frequency a constant parameter, the most common approach, and a dynamic decoupler based one, where the true system’s frequency, estimated by a PLL, is used by the controller.

In the first case, when a static decoupler is considered, it has been found from the study of the eigenvalues of a representative system that instabilities do not appear in power systems with normal frequency variations (8 Hz range), although a poor dynamic behavior is obtained. Nevertheless, the results show that wide frequency variations lead to control system instability if the frequency is sufficiently increased. Indeed, for the parameters set under study, the critical frequency results were equal to 81.7 Hz. Furthermore, if an error exists in the parameters of the system, the critical frequency is further lowered. For instance, if the filter inductor is increased to 130%, the critical frequency is reduced to 62.4 Hz, i.e., only a 12.4 Hz tolerance. In other words, a static decoupler shows a limited stable operating region when frequency variations are expected.

On the other hand, the dynamic decoupler-based controller uses the frequency information from the PLL to increase the allowable frequency operating range. The results show that for our system under study, the ac mains frequency may even go twice the nominal one (from 50 Hz up to 100 Hz), while the system remains stable. Thus, the frequency operating region is substantially increased, which is a key advantage over the static decoupler for these power systems. From the presented results, it can be concluded that power converters controllers must be carefully designed when supply frequency variations are expected, taking into account the expected variations in the grid frequency, as has been shown. To this end, the presented methodology can be a useful tool for the evaluation of the controller’s behavior.

## Figures and Tables

**Table 1:** System parameters.

Parameters	Value	P.U.
*v_g_*	220 V, rms	1
*v^dc^*	750 V	3.4
*Z_L_* (50 Hz)	8.8 Ω	1
*R_L_* (50 Hz)	7 Ω	0.80
*L_L_* (50 Hz)	17 mH	0.61
*R_g_* (50 Hz)	0.1 Ω	0.011
*L_g_* (50 Hz)	12 mH	0.43
*C_dc_* (50 Hz)	4.7 mF	0.36
*f_g_*	50 Hz	1
*f_sw_*	1.05 kHz	21
*N*	204	--
*k_c_* (current)	1.0	--
*T_i_* (current)	20 m	--
*k_c_* (*dc* voltage)	0.070	--
*T_i_* (*dc* voltage)	0.10	--

**Table 2:** Critical frequency for static decoupler.

Parameters	Value	Critical Frequency
*L_g_*	70%	Over 100 Hz
	nominal	81.7 Hz
	130%	62.4 Hz
*R_g_*	70%	81.2 Hz
	nominal	81.7 Hz
	130%	82.2 Hz
*C_dc_*	70%	82.4 Hz
	nominal	81.7 Hz
	130%	81.1 Hz

**Table 3:** Comparison of key features of *static* and *dynamic decoupler* based controllers.

Controller Algorithm	Robustness to Frequency Variation	Computational Burden	Dynamic Performance	Maintains Tuned Characteristics	Uses Frequency Value
*Static decoupler* based	Med	Low	Low	No	No
*Dynamic decoupler* based	High	Med	High	Yes	Yes

## Data Availability

Not applicable.
